# Feasibility study: spot-scanning proton arc therapy (SPArc) for left-sided whole breast radiotherapy

**DOI:** 10.1186/s13014-020-01676-3

**Published:** 2020-10-07

**Authors:** Sheng Chang, Gang Liu, Lewei Zhao, Joshua T. Dilworth, Weili Zheng, Saada Jawad, Di Yan, Peter Chen, Craig Stevens, Peyman Kabolizadeh, Xiaoqiang Li, Xuanfeng Ding

**Affiliations:** 1 Department of Radiation Oncology, Renmin Hospital, Wuhan University, Wuhan, 430060 Hubei Province China; 2 Department of Radiation Oncology, Beaumont Health System, Royal Oak, MI 48074 USA; 3 Cancer Center, Union Hospital, Tongji Medical College, Huazhong, University of Science and Technology, Wuhan, 430023 China; 4 School of Physics and Technology, Wuhan University, Wuhan, 430072 Hubei China

**Keywords:** Spot scanning, Proton arc, Left-sided breast cancer, Robust optimization

## Abstract

**Background:**

This study investigated the feasibility and potential clinical benefit of utilizing a new proton treatment technique: Spot-scanning proton arc (SPArc) therapy for left-sided whole breast radiotherapy (WBRT) to further reduce radiation dose to healthy tissue and mitigate the probability of normal tissue complications compared to conventional intensity modulated proton therapy (IMPT).

**Methods:**

Eight patients diagnosed with left-sided breast cancer and treated with breast-preserving surgery followed by whole breast irradiation without regional nodal irradiation were included in this retrospective planning. Two proton treatment plans were generated for each patient: vertical intensity-modulated proton therapy used for clinical treatment (vIMPT, gantry angle 10°–30°) and SPArc for comparison purpose. Both SPArc and vIMPT plans were optimized using the robust optimization of ± 3.5% range and 5 mm setup uncertainties. Root-mean-square deviation dose (RMSD) volume histograms were used for plan robustness evaluation. All dosimetric results were evaluated based on dose-volume histograms (DVH), and the interplay effect was evaluated based on the accumulation of single-fraction 4D dynamic dose on CT50. The treatment beam delivery time was simulated based on a gantry rotation with energy-layer-switching-time (ELST) from 0.2 to 5 s.

**Results:**

The average D1 to the heart and LAD were reduced to 53.63 cGy and 82.25 cGy compared with vIMPT 110.38 cGy (*p* = 0.001) and 170.38 cGy (*p* = 0.001), respectively. The average V5Gy and V20Gy of ipsilateral lung was reduced to 16.77% and 3.07% compared to vIMPT 25.56% (*p* = 0.001) and 4.68% (*p* = 0.003). Skin3mm mean and maximum dose were reduced to 3999.38 cGy and 4395.63 cGy compared to vIMPT 4104.25 cGy (*p* = 0.039) and 4411.63 cGy (*p* = 0.043), respectively. A significant relative risk reduction (RNTCP = NTCP_SPArc_/NTCP_vIMPT_) for organs at risk (OARs) was obtained with SPArc ranging from 0.61 to 0.86 depending on the clinical endpoint. The RMSD volume histogram (RVH) analysis shows SPArc provided better plan robustness in OARs sparing, including the heart, LAD, ipsilateral lung, and skin. The average estimated treatment beam delivery times were comparable to vIMPT plans when the ELST is about 0.5 s.

**Conclusion:**

SPArc technique can further reduce dose delivered to OARs and the probability of normal tissue complications in patients treated for left-sided WBRT.

## Introduction

Breast cancer is one of the most common cancers among women globally [1]. Breast-conserving surgery with adjuvant whole breast irradiation has become an increasingly popular treatment option for early-stage breast cancer [2–6]. Currently, conventional photon treatment methods such as tangential intensity-modulated radiation therapy (IMRT) and volumetric-modulated arc therapy (VMAT) have offered increased feasibility for normal tissue sparing in left-sided breast irradiation [7–9]. However, long-term follow-up data after adjuvant radiotherapy have shown increased risks of ischemic heart disease, presumably due to incidental irradiation of the heart. Left-sided WBRT involves closer proximity between the heart and radiation field and is associated with an increased rate of fatal cardiovascular events compared with women who received right-sided irradiation [5, 6, 10, 11]. Part of the anterior heart and left anterior descending artery (LAD) may receive significant dose during irradiation of the left-sided breast, and this may contribute to myocardial or coronary artery disease [12]. Darby et al. showed linear correlation between increasing mean heart dose and the incidence of ischemic heart disease among breast cancer patients [13]. Additionally, similar studies have shown that breast cancer patients are at a higher risk of long-term cardiac morbidity after radiation therapy treatment, which is directly related to the volume of the irradiated heart [5, 6]. Therefore, the optimization of WBRT has given increasing emphasis on reducing the cardiac dose.

Compared to photon radiotherapy, proton beam therapy may provide a dosimetric advantage when treating left-side breast cancer due to the sharp distal dose fall-off of the proton beam. Utilization of intensity modulated proton therapy (IMPT) for breast cancer treatment has increased over the last several years [14–16]. In IMPT, the positions and number of beam spots are optimized simultaneously to obtain the desired dose distribution, and robust optimization has been used to deal with uncertainties such as setup uncertainty, range uncertainty, and breathing motion uncertainty [17–22]. However, due to the low delivery efficiency with the current proton system, IMPT plans in breast cancer are still limited to a few beam angles. In addition, a large volume of the target may exceed the maximum field size. As a result, some IMPT plans may require a second isocenter for field matching [23], which further prolongs treatment time. These obstacles restrict the ability to further exploit the benefits of IMPT, and motivates us to explore better planning techniques to overcome the current limitations in terms of plan quality and clinical workflow efficiency. Spot-scanning proton arc therapy (SPArc) is an emerging technique that is able to deliver the proton beam through a dynamic rotational gantry [24]. Preliminary results demonstrated the potential clinical benefits for various disease sites, including prostate, head and neck, lung, and brain cancers [25–28]. This study is the first to exploit the feasibility and potential benefits of utilizing SPArc in the treatment of left-sided breast cancer patients compared to the conventional IMPT technique.

## Methods

### Retrospective patient data selection and treatment planning

Eight patients treated with whole breast irradiation without regional nodal irradiation from our institution using IMPT were included in this study. All patients underwent 4D-CT simulation using a spiral CT scanner (Philips Brilliance Big Bore, Philips Healthcare System, Cleveland, OH), and an average CT image was reconstructed based on a pixel-by-pixel averaging of the 4D-CT scan. The CT datasets were then transferred to RayStation version 9A (RaySearch Laboratories AB, Stockholm, Sweden) for planning. Clinical target volume (CTV) was defined as the volume irradiated based on the Radiation Therapy Oncology Group (RTOG) guidelines [29]. The internal target volume (ITV) was generated on the average CT scan, which was the union of the CTVs from all individual respiratory phase CT scans. Two separate treatment plans were created for each case: vertical IMPT (vIMPT, 10°-30°) and SPArc (partial-arc, 320°–150°) plans. Three of the patients with large tumors required two-isocenter IMPT plan due to the field size limitation (20 cm × 24 cm maximum field size). SPArc plans used a single isocenter with a partial arc. Both planning strategies used ITV plus robust optimization to take into account setup (± 5 mm) and range (± 3.5%) uncertainties (total 21 scenarios). The plan optimized using the Monte Carlo (MC) algorithm with a sampling history of 50,00 ions/spot, and a final dose computed using the MC algorithm with 1.0% statistical uncertainty and a dose grid of 3 mm. Proton beam model is based on the IBA ProteusONE energy range from 70 to 227 MeV, with spot size 1-sigma in air measurement ranging from 3.3 mm @227 MeV to 7.9 mm @70 MeV. The beam computation settings such as energy layer spacing and spot spacing were set by default in RayStation using automatic with scale 1 where Bragg peaks overlap at 80% of the max dose. Organs at risk (OARs) include heart, LAD, ipsilateral lung, contralateral breast and skin3mm. The skin3mm was defined as a 3 mm deep layer starting from the external body contour and following the extension of the ITV, and the ITV excludes the skin structure. The prescribed dose for all patients was 4256 cGy in 16 fractions [30, 31]. Plans aimed to achieve 100% of the prescribed dose in 98% of the ITV. SPArc and vIMPT plans were optimized in Raystation TPS in similar objectives and constraints for OARs. The objective and constrain functions were specified individually for each patient to obtain the best achievable treatment plan until there is no significant improvement.

### Nominal dosimetric plan quality evaluation and plan robustness analysis

Target coverage and doses to OAR’s were all evaluated and compared based on the DVH between SPArc and vIMPT. Also, the plan dose homogeneity was evaluated by homogeneity index (HI), which was defined as D_5_/D_95_ (where D5 and D95 are the minimum dose in 5% and 95% of the target volume). The ideal value of HI is 1. ITV coverage was evaluated by the conformality index (CI), which was defined as CI = (TVDp/TV)*(TVDp/VDp), where the TV is target volume, and TVDp and VDp are the target volume covered by the prescribed dose, and the volume enclosed by the prescription isodose line, respectively [32]. The plan robustness was defined by the ability of a proton plan to retain its objectives under the influence of uncertainties. In the present study, all plans were evaluated using the worst case scenario perturbed dose with setup uncertainties of ± 5 mm for x, y, z directions, and ± 3.5% range uncertainties. Besides, the root-mean-square deviation doses (RMSD) for each voxel of all the 21 scenarios were calculated. The RMSD volume histograms (RVH) and the area under the RVH curve (AUC), which introduced by Liu et al. were computed for relative comparison of IMPT and SPArc plan robustness [33]. The smaller the AUC value, the more robust the plan was for the specific structure(s).

### Evaluation of motion interplay effect

The interplay effect was evaluated by the single-fraction 4D dynamic dose calculation without considering re-scanning for different starting respiratory phases [34]. The 4D dynamic evaluation method distributes the spots over the different breathing cycle phases based on the delivery time and sequence. Then, the dose on each breathing phase were computed. Displacement vector fileds (DVFs) was generated via deformable image registation on the corresponding respiration phase to the reference 4D-CT phase (e.g.50% at this study). By utilizing the corresponding DVFs, the dose in each phase was mapped to the reference phase. The accumulation of the dose from different phases to the reference phase is called 4D dynamic dose [27]. It is assumed that the energy-layer-switching-time (ELST) of 1 s and a regular respiratory breathing cycle of 4.5 s in the study. The 4D dynamic dose calculation used a method by relating the time sequence of each spot delivery to the corresponding 4D-CT phase from the patient breathing cycle. Then it accumulated each spot dose via the deformable image registration on the corresponding respiration phase to the reference 4D-CT phase (CT50) associated with the corresponding DVF for evaluation.

### Treatment beam delivery time calculation and statistics analysis

The treatment delivery efficiency of SPArc and vIMPT plans were evaluated based on assumptions of a gantry with 1 rotation per minute gantry speed, 2 ms spot switching time, and ELST from 0.2 to 5 s [24]. Statistical analysis was performed with non-parametric Wilcoxon signed rank test using SPSS 21.0 software (International Business Machines, Armonk, New York). The *p* value < 0.05 was considered statistically significant.

### Evaluation of Potential clinical benefit for OARs based on the NCTP model

Potential clinical benefits of each OAR such as heart, LAD, left lung, and skin were estimated using the normal tissue complication probability (NTCP) model from the literature (Table 1). Briefly, Lyman–Kutcher–Burman (LKB) and Poisson LQ models were employed [35–39]. To compare risk values between SPArc and vIMPT plans, we defined the ratio of NTCP (R_NTCP_), as R_NTCP_ = NTCP_SPArc_/NTCP_vIMPT_.

**Table 1:** OARs, corresponding clinical endpoints, and NTCP models used in the present work

OAR	Clinical endpoint	References	Model
Heart	Mortality	Gagliardi et al. [35, 39]	Poission LQ model:D50 = 52.4 Gy,γ = 1.28,s = 1
LAD	Mortality	Gagliardi et al. [35, 39]	Poission LQ model:D50 = 52.4 Gy,γ = 1.28,s = 1
Left lung	Radiation pneumonitis	Seppenwoolde et al. [37, 38]	LKB model: TD50 = 30.8 Gy, m = 0.37, n = 0.99
Skin	Severe acute toxicity	Pastore et al. [36, 38]	LKB model: TD50 = 39 Gy, m = 0.14, n = 0.38

LKB model: $\mathrm{NTCP}=\frac{1}{\sqrt{2\pi }}{\int }_{-\infty }^{t}{e}^{{-t}^{2}/2}dt$, t = (D-TD_50_(V))/(m·TD_50_(V)), TD_50_(V) = TD_50_(1)/V^n^

Poission LQ model: $NTCP$=${\left\{1-\prod_{i=1}^{n}{\left[1-P{({D}_{i})}^{s}\right]}^{{V}_{i}/V}\right\}}^{1/s}$, $P$(${D}_{i}$) = ${2}^{-exp\left\{e\gamma (1-{D}_{i}/{D}_{50})\right\}}$

## Results

### Nominal dosimetric plan quality comparisons

**Fig. 1:**
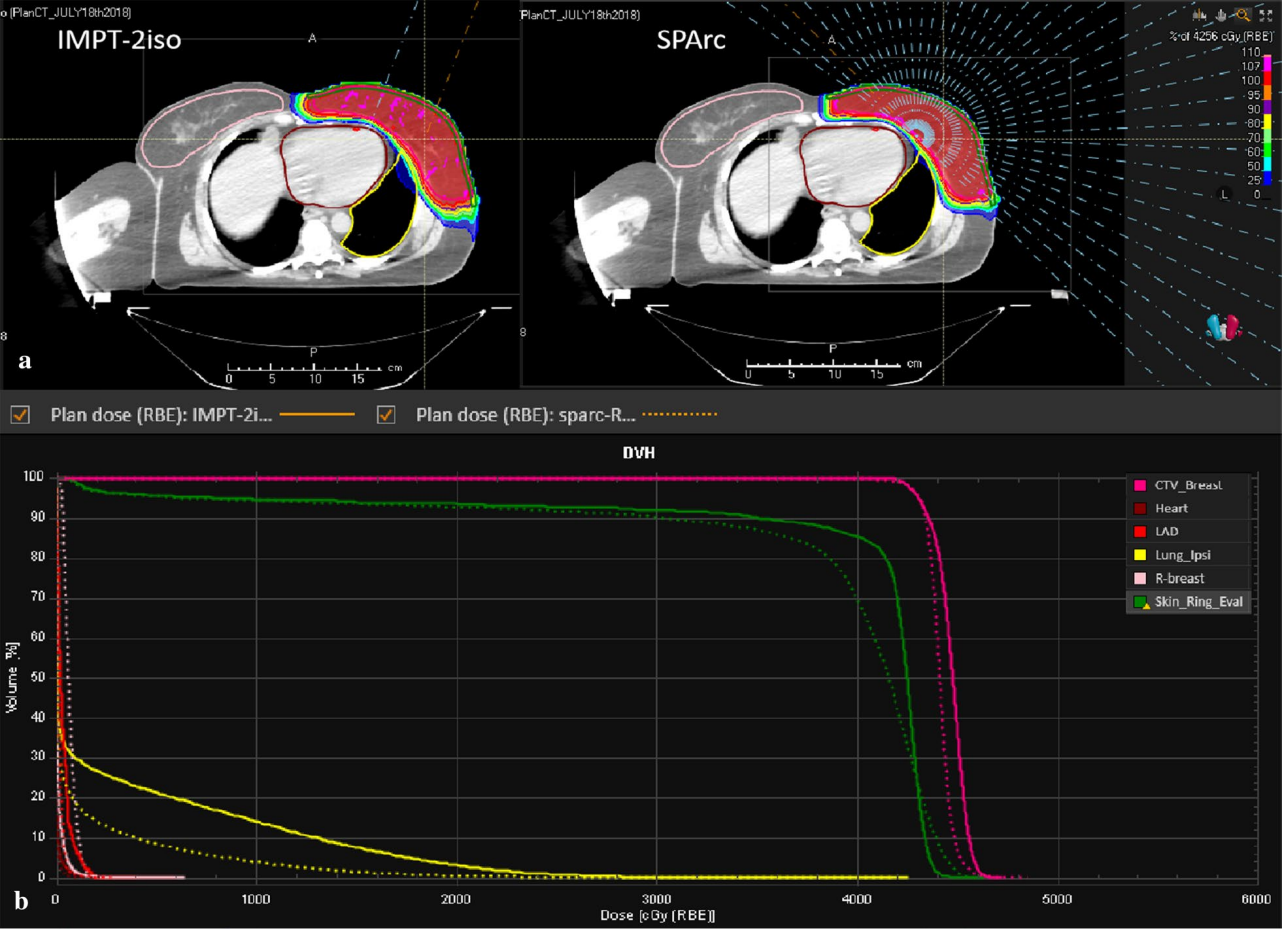
A representative of the radiation treatment plan from case #5. The comparison of **a** patient dose distribution, beam angle and **b** dose volume histograms (DVHs) (solid and dash lines for vIMPT and SPArc)

Figure 1 shows an example (patient #5) of radiation treatment plans and DVHs for SPArc and vIMPT. With a similar target coverage (Table 2), the SPArc technique achieved significantly higher dose homogeneity compared with the vIMPT technique (*p* = 0.005). Specifically, SPArc plans showed a significant reduction in heart dose (D1) of 51.42% compared to vIMPT (53.63 cGy vs 110.38 cGy, *p* = 0.001), as well as a substantial decrease in the maximum dose to LAD of 51.72% (82.25 cGy vs 170.38 cGy, *p* = 0.001). Compared to vIMPT, the volume of left lung received 500 (cGy) and 2000 (cGy) was reduced by 34.40% (16.77% vs 25.56%, *p* = 0.001) and 34.51% (3.07% vs 4.68%, *p* = 0.003) via SPArc. The skin3mm structure mean and maximum dose was reduced to 3999.38 cGy and 4395.63 cGy compared to vIMPT plans (4104.25 cGy (*p* = 0.039) and 4411.63 cGy (*p* = 0.043) respectively. However, the study found that the mean dose of the contralateral breast was increased to 18.5 cGy in the SPArc plans compared to the vIMPT plans (12.13 cGy, *p* = 0.011).

**Table 2:** Target volume and OARs dosimetric parameters for vIMPT and SPArc

Structure	Value	SPArc			vIMPT			Absolute difference (vIMPT − SPArc)		
		Planned	4D dynamic dose	AUC	Planned	4D dynamic dose	AUC	Planned	4D dynamic dose	AUC
ITV	D98(cGy)	4256	4262.75 ± 3.62	64.5 ± 15.55	4256	4265.63 ± 7.89	64.5 ± 5.73	–	2.88 ± 9.89 (*p* = 0.438)	0 ± 13.54 (*p* = 1)
HI		1.05 ± 0.01	1.05 ± 0.01		1.08 ± 0.02	1.07 ± 0.02		0.03 ± 0.02 (*p* = 0.005)	0.03 ± 0.02 (*p* = 0.006)	
CI		0.78 ± 0.06	0.79 ± 0.05		0.77 ± 0.06	0.77 ± 0.05		− 0.02 ± 0.02 (*p* = 0.044)	− 0.02 ± 0.02 (*p* = 0.018)	
heart	D1(cGy)	53.63 ± 18.19	56.88 ± 19.90	2.25 ± 0.89	110.38 ± 18.93	126.88 ± 26.25	4.00 ± 1.51	56.75 ± 34.08 (*p* = 0.001)	70.00 ± 32.14 (*p* < 0.001)	1.75 ± 1.39 (*p* = 0.009)
	Mean Dose(cGy)	4.5 ± 2.33	4.75 ± 2.31		6.38 ± 2.13	6.75 ± 2.05		1.88 ± 2.10 (*p* = 0.04)	2.00 ± 2.07 (*p* = 0.029)	
LAD	D1(cGy)	82.25 ± 37.38	87.38 ± 40.29	9.88 ± 3.68	170.38 ± 74.31	196.5 ± 61.57	21.25 ± 10.35	88.13 ± 49.66 (*p* = 0.001)	109.13 ± 57.17 (*p* = 0.001)	11.38 ± 9.29 (*p* = 0.01)
contralateral breast	Mean Dose(cGy)	18.5 ± 7.07	19.75 ± 8.01	4.75 ± 2.31	12.13 ± 2.70	11.88 ± 3.40	3.63 ± 2.45	− 6.37 ± 5.89 (*p* = 0.011)	− 7.88 ± 6.73 (*p* = 0.013)	− 1.13 ± 2.53 (*p* = 0.249)
ipsilateral lung	V500(cGy)	16.77 ± 7.18	16.63 ± 7.13	122.63 ± 38.26	25.56 ± 5.95	25.73 ± 5.27	168.25 ± 29.05	8.79 ± 5.25 (*p* = 0.001)	9.11 ± 5.28 (*p* = 0.002)	45.63 ± 21.54 (*p* = 0.001)
	V2000(cGy)	3.07 ± 2.17	3.06 ± 2.12		4.68 ± 1.78	4.67 ± 1.77		1.61 ± 1.03 (*p* = 0.003)	1.61 ± 1.04 (*p* = 0.003)	
	Mean dose(cGy)	282.75 ± 128.73	280.29 ± 127.59		395.38 ± 91.19	400.69 ± 94.82		112.63 ± 88.06 (*p* = 0.009)	120.40 ± 77.92 (*p* = 0.003)	
Skin3mm	D1(cGy)	4395.63 ± 98.35	4386.25 ± 96.62	85.5 ± 11.71	4411.63 ± 72.03	4402.5 ± 107.87	81.25 ± 27.73	16.00 ± 113.86 (*p* = 0.043)	16.25 ± 113.92 (*p* = 0.05)	− 4.25 ± 21.10 (*p* = 0.587)
	Mean Dose(cGy)	3999.38 ± 120.57	3992 ± 108.02		4104.25 ± 110.34	4097.75 ± 90.84		104.87 ± 115.17 (*p* = 0.039)	105.75 ± 112.03 (*p* = 0.032)	

*ITV* internal target volume, *HI* homogeneity index, *AUC* area under the curve

### Plan robustness evaluation in the presence of the setup and range uncertainties

**Fig. 2:**
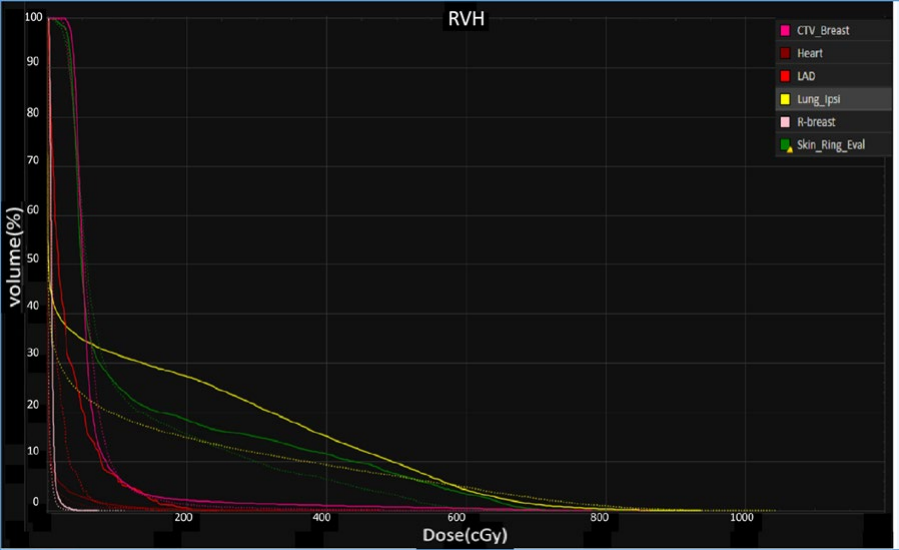
Root-mean square dose volume histogram (RVH) of different OARs. The solid line is vIMPT and the dashed line is SPArc (case #5)

All the AUC values of target volumes and OARs from eight cases were evaluated. With a comparable target coverage, some dosimetric impacts of OARs were mitigated in the presence of setup and range errors via SPArc compared to vIMPT, such as heart (4.00 in vIMPT plan versus 2.25 in SPArc plan, *p* = 0.009), left-lung (168.25 in vIMPT versus 122.63 in SPArc, *p* = 0.001) and LAD (21.25 in vIMPT versus 9.88 in SPArc, *p* = 0.01). There is no statistical difference in contralateral-breast and skin3mm’s dosimetric robustness. Figure 2 illustrates RVHs from case number 5.

### Evaluation of dosimetric impact from the interplay effect

**Fig. 3:**
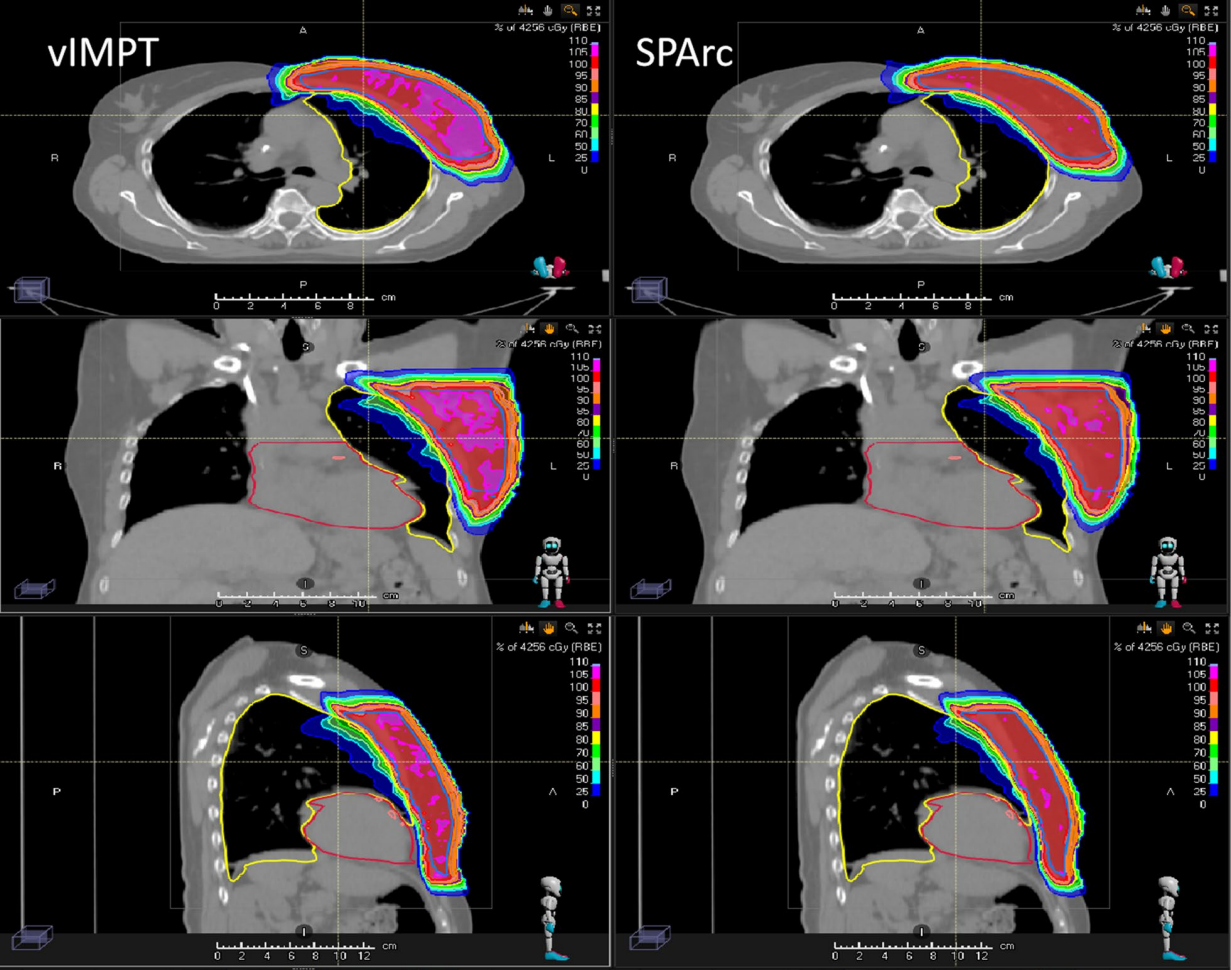
The single-fraction 4D dynamic dose distributions on phase (CT50) for vIMPT and SPArc

The study found that SPArc could improve the ability to mitigate the interplay effect in both target and OARs (Table 2), where the SPArc was able to maintain the HI of dose (1.05 vs 1.07, *p* = 0.006) and the CI of ITV (0.79 vs 0.77, *p* = 0.018). In addition, SPAc was able to mitigate the dose variation such as D1 of heart (on average increased 3.25 cGy) compared to single field IMPT (on average increased 16.50 cGy), and D1 of LAD (on average increased 5.13 cGy) compared to single field IMPT (on average increased 26.12 cGy). Figure 3 shows a representative example of the 4D dynamic dose calculation of SPArc versus vIMPT plans.

### Beam delivery efficiency

**Fig. 4:**
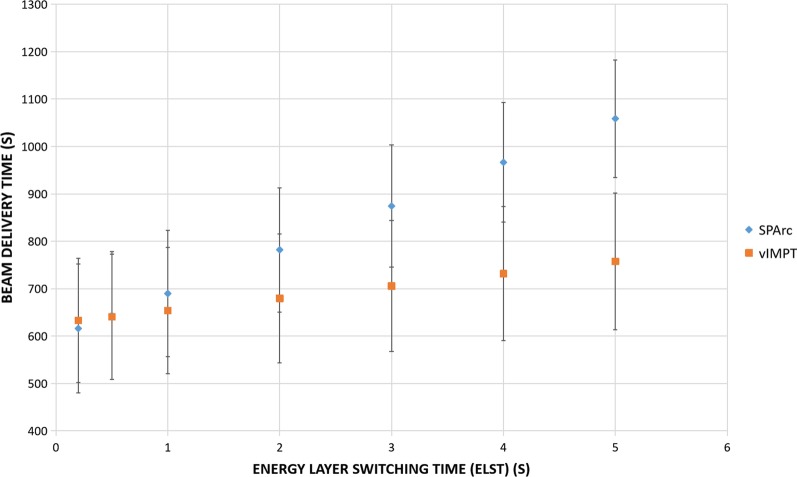
Total average treatment beam delivery time

Table 3 lists the estimated beam delivery time per fraction for both SPArc and vIMPT plans for various ELST. When the proton system’s ELST is 5 s, the average estimated delivery time ratios between SPArc and vIMPT plans was 1.40 (1059 s vs. 758 s), which means it would take significantly longer to deliver a SPArc plan (*p* < 0.001). The difference became smaller as the ELST is faster. When the ELST was less than 0.5 s, the treatment delivery time of SPArc plan could be less than vIMPT (*p* = 0.005) (Fig. 4). However, the estimated treatment time did not take into account the additional time to perform iso-shift and re-imaging. For the 2-isoenter vIMPT plan, additional couch movement for the next iso and IGRT verification procedures may be needed to ensure the treatment accuracy. For SPArc, only a single iso is needed, which would save significant additional treatment time as well as simplify the clinical treatment workflow.

**Table 3:** Plan parameter comparison between vIMPT and SPArc

Plan parameters	vIMPT	SPArc	Absolute difference (SPArc − vIMPT)
Beam directions	1	39	38
Total energy layers	27 ± 3.85	93 ± 4.57	66 ± 6.95
Total monitor unit	6143 ± 1281.08	5511 ± 1233.95	− 633 ± 140.91
Total delivery time (5 s)	758 ± 144.17	1059 ± 123.77	301 ± 30.34 (*p* < 0.001)
Total delivery time (4 s)	732 ± 141.3	967 ± 126.08	235 ± 24.17 (*p* < 0.001)
Total delivery time (3 s)	706 ± 138.53	874 ± 128.50	169 ± 18.41 (*p* < 0.001)
Total delivery time (2 s)	680 ± 135.79	782 ± 131.04	102 ± 13.64 (*p* < 0.001)
Total delivery time (1 s)	654 ± 133.11	690 ± 133.68	36 ± 11.18 (*p* < 0.001)
Total delivery time (0.5 s)	641 ± 131.79	644 ± 135.03	3 ± 11.35 (*p* = 0.47)
Total delivery time (0.2 s)	633 ± 131.00	616 ± 135.86	− 17 ± 11.93 (*p* = 0.005)

### Potential clinical benefit for heart

**Fig. 5:**
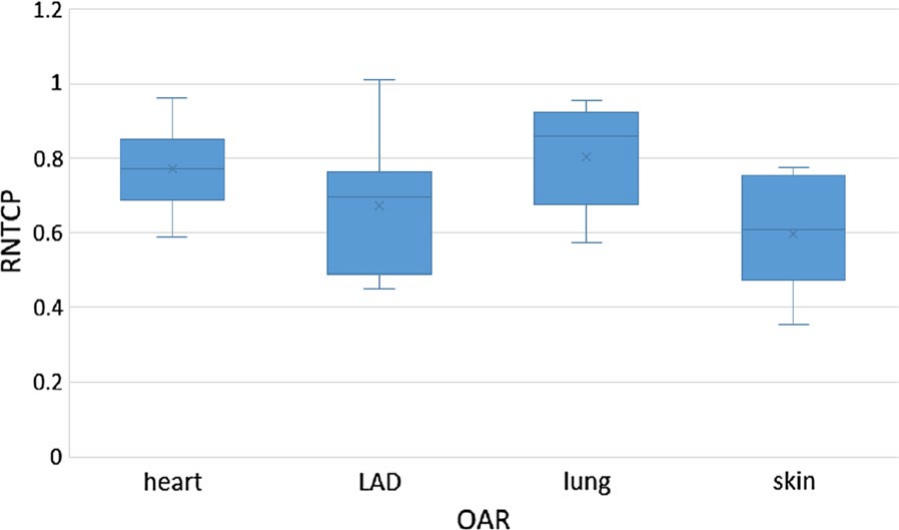
Box-whisker plot of RNTCP comparison according to NTCP analysis for heart, LAD, skin and lung

The results show that there was a potential clinical benefit based on NTCP model calculation of using SPArc over vIMPT (Table 4). More specifically, heart, LAD, left-lung, and skin complications showed an overall reduction in the toxicity risk prediction for SPArc plans compared with the vIMPT plan, with R_NTCP_ ranging from 0.61 to 0.86, depending on the clinical endpoint (Fig. 5).

**Table 4:** R_NTCP_ ratio comparison according to normal tissue complication probability (NTCP) analysis for heart, LAD, skin and lung

		Median (range)	
OAR	Clinical endpoint	R_NTCP_ = NTCP_SPArc_/NTCP_vIMPT_	*p* value
Heart	Major coronary events	0.77 (0.59–0.96)	0.003
LAD	Coronary stenosis	0.69 (0.45–1.01)	0.119
Left lung	Radiation pneumonitis	0.86 (0.57–0.95)	0.005
Skin	Severe acute toxicity	0.61 (0.35–0.78)	0.007

## Discussion

This is a first and comprehensive dosimetric planning study to explore the feasibility and potential dosimetric and clinical benefits in the management of patients with left-sided breast cancer receiving whole breast irradiation. This study also analyzed plan robustness in the presence of setup and range errors in addition to the breathing-induced interplay effect. Our results indicate that the SPArc technique with additional degree of freedom in optimization and delivery could not only improve dosimetric quality but also improve plan robustness compared to conventional vIMPT. Recently, there is a trend to use more fields in the breast cancer treatment which might be able to improve the treatment plan quality as well. To provide a more comprehensive comparison among these planning strategies, additional data were included in the Additional file 1 including the comparison with 3F-IMPT and 5F-IMPT. The result showed that as more beam angles were used in IMPT, the more robust the plan quality is. However, as a tradeoff, multi-field IMPT takes longer to deliver.

In addition to the plan quality improvement, one of the driven motivation of SPArc is to shorten the treatment delivery time and simplify the clinical workflow. The results from this study agree with previous findings that SPArc could shorten the total treatment delivery time based on the modern proton therapy machines where the average of ELST is less than 0.5 s [25–28, 40]. In the presence of the large target size, which requires multi-isoenter field matching, SPArc technique could utilize a single-isoenter to simplify the clinical treatment workflow. This is due to the current *en face* beam angle selection. A 2-iso setup was needed where the target exceeds the lateral maximum field size. e.g. for IBA ProteusONE, the lateral max field size is 20 cm. Any target which was larger than 20 cm laterally from Beam-Eye-View, requires additional iso. By taking advantage of the arc trajectory, SPArc can deliver the proton spot to the boundary of the lateral region through a tangent beam direction. Thus, SPArc effectively increased the lateral target coverage by using the single iso. Such principle also applies for multi-field IMPT e.g. 3F-IMPT and 5F-IMPT where single-iso setup was needed. However, please be aware of that SPArc or multi-field IMPT will not solve the problem where the target exceed the max field size in superior-inferior direction. In these scenarios, multi-iso setups for SPArc are still needed. For example, three out of eight cases included in this study required a second isocenter. As a result, therapists need to apply an isocenter shift, image validation, and second treatment field in the vIMPT treatment. A review of treatment logs of these three cases found that it took 5.11 ± 0.05 min on average to perform these additional procedures for the 2^nd^ isocenter shift. These additional couch isocenter shift and image acquisition times prolong the overall treatment time and also increase the chance of intrafraction motion [41–43]. Thus, SPArc has the potential to provide a more efficient clinical treatment workflow through one arc trajectory and further reduce the uncertainties from the intrafraction motion.

Cardiac toxicity remains a leading treatment related cause of morbidity and mortality among long-term breast cancer survivors after radiotherapy, especially in the patient population with left-sided breast cancer [44]. Previous studies have found several heart dosimetric metrics related to acute or late cardiotoxicity, although there are still debates in which dosimetric metric and substructures are more related to the acute or late cardiotoxicity [45–48].

Darby et al. found that the rate of the incidence of ischemic heart disease increased linearly with the mean heart dose by 7.4% per Gy [13]. In addition, the RADCOMP (Radiotherapy Comparative Effectiveness) trial has also pointed out that the mean heart dose as a critical indicator for cardiotoxicity [45, 49]. The mean heart dose of the delivered vIMPT plans in our study was 6.38 cGy, which is higher than SPArc 4.5 cGy (*p* = 0.04). Moreover, there is increasing evidence that the dose of heart substructures needs to be considered. Some studies have focused on the LAD as important parts of the heart associated with radiation-induced heart disease [11, 50]. Conventional proton beam therapy (IMPT or Passive-scattering) could reduce the dose of the heart and LAD in left-side breast cancer patients compared to the photon radiotherapy technique in the high cardiac doses sparing [10, 15, 51]. This study found that the new proton treatment technique, SPArc, could further reduce the D1 of heart and LAD which might mitigate the probability of heart acute and late toxicities. We recognize that the relevance of photon NTCP models to proton therapy has not been established and further proton study would be needed to correlate the proton dose with the cardiotoxicity. The study also found that the contralateral breast mean doses were slightly higher in SPArc planning group compared with vIMPT. It is important to consider and choose the optimal treatment technology for an individual patient considering the possible clinical benefits as well as the limitation of using SPArc technique.

Another critical OAR that could benefit from SPArc is the healthy lung tissue. Reducing the radiation dose to the lung can result in reducing the risk of radiation pneumonitis in patients. Our feasibility study finds that the technology of SPArc can substantially improve not only the heart and LAD sparing but also the lung sparing in comparison with vIMPT. Previous studies have confirmed that proton therapy can significantly reduce the V500(cGy) and V2000(cGy) of the ipsilateral lung by nearly 50% compared to traditional 3DCRT and IMRT [10, 52, 53]. This study found that SPArc plans further reduced all dose-volume parameters while providing a reduced or similarly high-dose radiation volume with IMPT in left-sided WBRT.

The study showed a very interesting result where SPArc has better capability of mitigating the motion interplay effect over IMPT, even though SPArc deliver spots through some tangent arc trajectories which are supposed to be more sensitive to the motion and it has a similar treatment delivery time compared to the single field IMPT. Although the exact rationale behind this phenomenon of interplay effect mitigation is not well understood yet, a similar finding was also reported in the lung mobile target treatment in comparison between SPArc and IMPT [27]. There might have one hypothesis to explain the phenomena. When the number of beam angles increases, it could effectively reduce the dosimetric impact from the proton range uncertainties. For example, when the tumor moves in and out the beamline due to the breathing induce motion, there might have 50% of dose overshooting or undershooting the target from each beam angles using a two-field IMPT plan. On the other hand, SPArc, as an advanced IMPT technique consists of hundreds of beam angles. As a result, overshooting or undershooting the target might only contribute a few percentages of total dose difference in each beam angle. Such advantage may help SPArc effectively mitigate the dosimetric impact from the interplay effect. Because the breathing-induced motion is not significant (< 2 mm, Additional file 1: Table S4.) in most of the breast cancer patient population, it is limitation of this motion evaluation study. To prove this new hypothesis of interplay mitigation effect in a relationship to the degree of freedom or beam angles, a more quantitative study would be needed.

Besides, spot characteristics also play an important role in the interplay effect evaluation [54]. In addition, the spot spacing parameter for planning optimization determine the number of the spots where a higher value increases the inter-spot distance and less spot would be used in a plan. Thus, the plan might be more sensitive to the motion uncertainties [55, 56]. Similarly, the energy layer spacing parameter determine the number of energy layers [57]. These planning optimization parameters may also play a critical role in the interplay effect. We would recommend different institutions to evaluate the interplay effect based on their own proton beam model and planning optimization parameters in order to offer an optimal treatment plan with an efficiency delivery and robust plan quality [58].

## Conclusions

SPArc can achieve superior OARs sparing and robust plan quality in left-sided WBRT compared to the traditional IMPT. With ELST less than 0.5 s in current modern proton systems, the total beam delivery time per fraction of SPArc would be faster than IMPT which would be desirable for future clinical implementation.

## Supplementary information

**Additional file 1:** A comprehensive comparison of SPArc, vIMPT, 3F-IMPT, 5F-IMPT in terms of the plan quality, robustness evaluation and treatment delivery efficiency. **Table s1.** Target volume and OARs dosimetric parameters among vIMPT, 3F-IMPT, 5F-IMPT and SPArc. **Table s2.** Absolute difference of target volume and OARs dosimetric parameters to SPArc. **Table s3.** Absolute difference of target volume and OARs dosimetric parameters to vIMPT. **Figure s1.** Total average treatment beam delivery time. **Table s4.** The movement was calculated based on the mass centre difference between the CTVs in the 4DCT phases in 3D, superior inferior (SI), left-right (LR) and anterior-posterior (AP).

## Data Availability

All data generated or analyzed during this study are included in this published article. Additional information is available from the corresponding author on reasonable request.

## Abbreviations

LAD: Left anterior descending
IMPT: Intensity modulated proton therapy
SPArc: Spot-scanning proton arc
vIMPT: Vertical intensity-modulated proton therapy
RMSD: Root-mean-square deviation dose
DVH: Dose volume histogram
ELST: Energy-layer-switching-time
D1: Dose received by 1% of volume
IMRT: Intensity modulated radiation therapy
VMAT: Volumetric modulated arc therapy
RTOG: Radiation therapy oncology group
ITV: Internal target volume
MC: Monte Carlo
HI: Homogeneity index
CI: Conformality index
D5: Dose received by 5% of volume
D95: Dose received by 95% of volume
RVH: RMSD volume histograms
AUC: Area under the RVH curve
OARs: Organ at risks
NTCP: Normal tissue complication probability
LKB: Lyman–Kutcher–Burman
RADCOMP: Radiotherapy comparative effectiveness
V500: Volume received at least 500 cGy
V2000: Volume received at least 2000 cGy
WBRT: Whole breast radiotherapy
