# Supplementary material for: Feasibility study: spot-scanning proton arc therapy (SPArc) for left-sided whole breast radiotherapy
Source: Radiat Oncol. 2020 Oct 7;15:232. doi: 10.1186/s13014-020-01676-3 (PMC7542109; doi:10.1186/s13014-020-01676-3)
Supplement: Supplementary file 1 — Additional file 1: A comprehensive comparison of SPArc, vIMPT, 3F-IMPT, 5F-IMPT in terms of the plan quality, robustness evaluation and treatment delivery efficiency. Table s1. Target volume and OARs dosimetric parameters among vIMPT, 3F-IMPT, 5F-IMPT and SPArc. Table s2. Absolute difference of target volume and OARs dosimetric parameters to SPArc. Table s3. Absolute difference of target volume and OARs dosimetric parameters to vIMPT. Figure s1. Total average treatment beam delivery time. Table s4. The movement was calculated based on the mass centre difference between the CTVs in the 4DCT phases in 3D, superior inferior (SI), left-right (LR) and anterior-posterior (AP). [file 13014_2020_1676_MOESM1_ESM.docx]

**Additional file 1**

Comparison of SPArc, vIMPT, 3F-IMPT, 5F-IMPT

The study has demonstrated that the new SPArc therapy technique has the potential dosimetric and clinical advantages for whole breast irradiation over the current clinical practice using a single *en face* or vertical field. As some proton clinics used more treatment fields, similar dosimetric advantage will be observed as well. SPArc is considered as an advanced IMPT with hundreds of beam angles. It would be interested to see the different between the multi-field IMPT vs SPArc.

To provide a comprehensive comparison over the vIMPT plan or SPArc plan, 3F-IMPT and 5F-IMPT planning groups were generated using a single-iso setup similar to SPArc. The same prescription and robust optimization parameter were used as described in the methods and materials. The result showed that with an equivalent target coverage in the nominal plan, 3F-IMPT and 5F-IMPT significant reduced heart D1 (95.00 vs 110.38, p=0.011; 82.75 vs 110.38, p=0.001) and LAD D1 (125.75 vs 170.38, p=0.022; 112.75 vs 170.38, p=0.004) compared to vIMPT (Table s1 & s3). Robustness evaluations showed that 3F and 5F IMPT significant improved the LAD (13.63 vs 21.25, p=0.035; 12.38 vs 21.25, p=0.019) and ipsilateral lung (106.13 vs 168.25, p<0.001; 101.13 vs 168.25, p<0.001) dosimetric robustmess compared to vIMPT (Table s1 & s3).

Compared to SPArc, 3F-IMPT and 5F-IMPT provided a comparable CI (0.77 and 0.78 vs 0.78), but the SPArc plan had clear advantages on the HI than 3F-IMPT and 5F-IMPT (1.07 and 1.07 vs 1.05). In addition, SPArc significantly reduced heart D1 and LAD D1 to 53.63 cGy and 82.25 cGy compared to 3F-IMPT 95.00cGy (p<0.001) and 125.75 cGy (p=0.001), and 5F-IMPT 82.75cGy (p=0.001) and 112.75 cGy (p=0.002). Some OARs dosimetric robustness were significantly improved with SPArc compared to 3F-IMPT and 5F-IMPT (Table s1 & s2) such as heart (3.88 and 3.13 in 3F-IMPT and 5F-IMPT plan versus 2.25 in SPArc plan, p=0.002 and p=0.006), and LAD (13.63 in 3F-IMPT versus 9.88 in SPArc, p=0.006). There is no statistical difference in contralateral breast, ipsilateral lung, and skin3mm’s dosimetric robustness. In conclusion, 3F/5F IMPT with increased degree of freedom did improve some dosimetric metrics over vIMPT. However, SPArc with hundreds of beam angles, is still superior to the 3F/5F IMPT in terms of plan quality.

Please be aware of that 3F/5F IMPT did improve some dosimetric metrics compared to the vIMPT. As a tradeoff, multi-field IMPT prolong the treatment delivery time because of the increased number of energy layers as well as spot numbers when the ELST is more than 1 second (Figure s1). However, there is no significant difference when the ELST is close to 1 s. The estimated treatment delivery time listed in the table s4 only include beam-on time and gantry rotational time, the beam request time and treatment field loading time from OIS e.g. ARIA or MOSIAQ to the proton delivery system was not taken into account. In other words, addition time is added into the clinical workflow in the multi-field IMPT treatment. This is one of the motivation that the proton treatment technique is moving towards the arc approach which not only shorten the treatment delivery time but also simplify the clinical treatment workflow.

**Table S1.** Target volume and OARs dosimetric parameters among vIMPT, 3F-IMPT, 5F-IMPT and SPArc

|  |  | 1F-IMPT(vIMPT) | | 3F-IMPT | | 5F-IMPT | | SPArc | |
| --- | --- | --- | --- | --- | --- | --- | --- | --- | --- |
| structure | value | Plan | AUC | Plan | AUC | Plan | AUC | Plan | AUC |
| ITV | D98(cGy) | 4256 | 64.5±5.73 | 4256 | 67.38±8.78 | 4256 | 65.63±12.14 | 4256 | 64.5±15.55 |
| HI |  | 1.08±0.02 | - | 1.07±0.01 | - | 1.07±0.01 | - | 1.05±0.01 | - |
| CI |  | 0.77±0.06 | - | 0.77±0.05 | - | 0.78±0.04 | - | 0.78±0.06 | - |
| Heart | D1(cGy) | 110.38±18.93 | 4.00±1.51 | 95.00±12.40 | 3.88±0.64 | 82.75±14.99 | 3.13±0.64 | 53.63±18.19 | 2.25±0.89 |
|  | Mean dose(cGy) | 6.38±2.13 | - | 5.75±1.83 | - | 5.25±1.28 | - | 4.5±2.33 | - |
| LAD | D1(cGy) | 170.38±74.31 | 21.25±10.35 | 125.75±40.39 | 13.63±3.62 | 112.75±46.72 | 12.38±4.72 | 82.25±37.38 | 9.88±3.68 |
| Contralateral breast | Mean dose(cGy) | 12.13±2.70 | 3.63±2.45 | 11.38±4.24 | 4.25±2.87 | 14.13±4.12 | 4.63±1.85 | 18.5±7.07 | 4.75±2.31 |
| Ipsilateral lung | V500(cGy) | 25.56±5.95 | 168.25±29.05 | 19.62±4.62 | 106.13±20.61 | 17.47±4.28 | 101.13±24.42 | 16.77±7.18 | 122.63±38.26 |
|  | V2000(cGy) | 4.68±1.78 | - | 3.39±1.99 | - | 3.00±2.14 | - | 3.07±2.17 | - |
|  | Mean dose(cGy) | 395.38±91.19 | - | 314.50±84.71 | - | 289.50±86.43 | - | 282.75±128.73 | - |
| Skin3mm | D1(cGy) | 4411.63±72.03 | 81.25±27.73 | 4432.25±80.93 | 112.88±13.60 | 4410.38±79.84 | 110.50±20.61 | 4395.63±98.35 | 85.5±11.71 |
|  | Mean dose(cGy) | 4104.25±110.34 | - | 4014.13±120.86 | - | 3983.88±132.57 | - | 3999.38±120.57 | - |

**Table S2.** Absolute difference of target volume and OARs dosimetric parameters to SPArc

| Absolute difference to SPArc | | | | | | | |
| --- | --- | --- | --- | --- | --- | --- | --- |
| structure | value | Plan(1F-IMPT) | AUC(1F-IMPT) | Plan(3F-IMPT) | AUC(3F-IMPT) | Plan(5F-IMPT) | AUC(5F-IMPT) |
| ITV | D98(cGy) | - | 0±13.54 (p=1) | - | -2.88±18.39 (p=0.672) | - | -1.13±9.99 (p=0.759) |
| HI |  | -0.03±0.02 (p=0.005) | - | -0.02±0.01 (p<0.001) | - | -0.02±0.01 (p<0.001) | - |
| CI |  | 0.02±0.02 (p=0.044) | - | 0.01±0.03 (p=0.311) | - | 0.01±0.03 (p=0.490) | - |
| Heart | D1(cGy) | -56.75±34.08 (p=0.001) | -1.75±1.39 (p=0.009) | -41.38±17.94 (p<0.001) | -1.63±0.92 (p=0.002) | -29.13±14.50 (p=0.001) | -0.88±0.64 (p=0.006) |
|  | Mean dose(cGy) | -1.88±2.10 (p=0.04) | - | -1.25±2.38 (p=0.15) | - | -0.75±1.98 (p=0.32) | - |
| LAD | D1(cGy) | -88.13±49.66 (p=0.001) | -11.38±9.29 (p=0.01) | -43.50±22.56 (p=0.001) | -3.75±2.71 (p=0.006) | -30.50±17.77 (p=0.002) | -2.50±3.34 (p=0.072) |
| Contralateral breast | Mean dose(cGy) | 6.37±5.89 (p=0.011) | 1.13±2.53 (p=0.049) | 7.13±4.73 (p=0.004) | 0.50±2.83 (p=0.632) | 4.38±4.81 (p=0.037) | 0.13±1.73 (p=0.844) |
| Ipsilateral lung | V500(cGy) | -8.79±5.25 (p=0.001) | -45.63±21.54 (p=0.001) | -2.86±4.13 (p=0.092) | 16.50±25.21 (p=0.107) | -0.71±3.87 (p=0.621) | 21.50±25.56 (p=0.049) |
|  | V2000(cGy) | -1.61±1.03 (p=0.003) | - | -0.32±0.38 (p=0.047) | - | 0.07±0.77 (p=0.807) | - |
|  | Mean dose(cGy) | -112.63±88.06 (p=0.009) | - | -31.75±50.19 (p=0.117) | - | -6.75±64.80 (p=0.777) | - |
| Skin3mm | D1(cGy) | -16.00±113.86 (p=0.043) | 4.25±21.10 (p=0.587) | -36.63±102.42 (p=0.345) | -27.38±15.46 (p=0.002) | -14.75±51.23 (p=0.442) | -25.00±20.61 (p=0.011) |
|  | Mean dose(cGy) | -104.87±115.17 (p=0.039) | - | -14.75±104.67 (p=0.702) | - | 3.00±76.62 (p=0.915) | - |

**Table S3.** Absolute difference of target volume and OARs dosimetric parameters to vIMPT

| Absolute difference to vIMPT | | | | | |
| --- | --- | --- | --- | --- | --- |
| structure | value | Plan(3F-IMPT) | AUC(3F-IMPT) | Plan(5F-IMPT) | AUC(5F-IMPT) |
| ITV | D98(cGy) | - | -2.88±12.69 (p=0.542) | - | -1.13±12.70 (p=0.809) |
| HI |  | 0.01±0.02(p=0.434) | - | 0.00±0.02(p=0.687) | - |
| CI |  | -0.01±0.04(p=0.666) | - | -0.01±0.03 (p=0.441) | - |
| Heart | D1(cGy) | 15.38±12.73 (p=0.011) | 0.13±1.36 (p=0.802) | 27.63±15.47 (p=0.001) | 0.88±1.36(p=0.111) |
|  | Mean dose(cGy) | -0.13±1.36 (p=0.802) | - | 0.38±2.45 (p=0.678) | - |
| LAD | D1(cGy) | 44.63±43.27 (p=0.022) | 7.63±8.26 (p=0.035) | 57.63±39.54 (p=0.004) | 8.88±8.24 (p=0.019) |
| Contralateral breast | Mean dose(cGy) | 0.75±4.40(p=0.644) | -0.63±3.85 (p=0.660) | -2.00±4.41(p=0.240) | -1.00±2.93 (p=0.366) |
| Ipsilateral lung | V500(cGy) | 5.94±4.54 (p=0.008) | 62.13±11.48 (p<0.001) | 8.09±5.64 (p=0.005) | 67.13±12.01 (p<0.001) |
|  | V2000(cGy) | 1.30±1.09(p=0.012) | - | 1.69±1.43 (p=0.013) | - |
|  | Mean dose(cGy) | 80.88±54.18 (p=0.004) | - | 105.88±81.73 (p=0.008) | - |
| Skin3mm | D1(cGy) | -20.63±110.96 (p=0.615) | -31.63±32.69 (p=0.029) | -1.25±108.26 (p=0.975) | -29.25±34.85 (p=0.049) |
|  | Mean dose(cGy) | 90.13±122.66(p=0.076) | - | 107.88±112.16(p=0.030) | - |

**Figure S1.** Total average treatment beam delivery time.

**Table S4.**The movement was calculated based on the mass centre difference between the CTVs in the 4DCT phases in 3D, superior inferior (SI), left-right (LR) and anterior-posterior (AP) .

| Patient | CTV mass centre movement in 3D (SI, LR, AP ) [mm] | | |
| --- | --- | --- | --- |
|  | SI | LR | AP |
| 1 | 0.5 | 0.6 | 0.1 |
| 2 | 0.8 | 0.3 | 0.7 |
| 3 | 0.6 | 0.9 | 0.5 |
| 4 | 1.3 | 0.1 | 1.2 |
| 5 | 0.5 | 1.2 | 0.8 |
| 6 | 2.9 | 0.8 | 0.7 |
| 7 | 0.3 | 0.7 | 0.6 |
| 8 | 1.4 | 0.9 | 1.2 |
| average | 1.04±0.85 | 0.69±0.35 | 0.73±0.36 |

Abbreviations: AP: anterior-posterior; LR: left-right; SI: superior-inferior.
